# Global residual stress field inference method for die-forging structural parts based on fusion of monitoring data and distribution prior

**DOI:** 10.1186/s42492-025-00187-w

**Published:** 2025-03-06

**Authors:** Shuyuan Chen, Yingguang Li, Changqing Liu, Zhiwei Zhao, Zhibin Chen, Xiao Liu

**Affiliations:** 1 College of Mechanical and Electrical Engineering, Nanjing University of Aeronautics and Astronautics, Nanjing, Jiangsu 210016 China; 2 School of Mechanical and Aerospace Engineering, Queen’s University Belfast, Belfast, BT9 5AH UK; 3 Shanghai Aerospace Equipment Manufacture Co., Ltd, Shanghai, 200245 China

**Keywords:** Die-forging structural parts, Global residual stress field inference, Residual stress field partitioning, Deformation force

## Abstract

Die-forging structural parts are widely used in the main load-bearing components of aircrafts because of their excellent mechanical properties and fatigue resistance. However, the forming and heat treatment processes of die-forging structural parts are complex, leading to high levels of internal stress and a complex distribution of residual stress fields (RSFs), which affect the deformation, fatigue life, and failure of structural parts throughout their lifecycles. Hence, the global RSF can provide the basis for process control. The existing RSF inference method based on deformation force data can utilize monitoring data to infer the global RSF of a regular part. However, owing to the irregular geometry of die-forging structural parts and the complexity of the RSF, it is challenging to solve ill-conditioned problems during the inference process, which makes it difficult to obtain the RSF accurately. This paper presents a global RSF inference method for the die-forging structural parts based on the fusion of monitoring data and distribution prior. Prior knowledge was derived from the RSF distribution trends obtained through finite element analysis. This enables the low-dimensional characterization of the RSF, reducing the number of parameters required to solve the equations. The effectiveness of this method was validated in both simulation and actual environments.

## Introduction

Die-forging structural parts are widely used in the primary load-bearing components of aircraft fuselages because of their large formable thickness, few internal defects, excellent mechanical properties, and long service life. However, during the forging and heat treatment processes, residual stress fields (RSFs) characterized by high stress levels and complex distributions are induced within die-forging blanks because of phase transformation and uneven temperature fields.

The residual stress is the internal stress within die-forging parts that remains in the material in self-equilibrium after eliminating external forces and thermal loads. During the manufacturing or service stages, the interaction between the RSF and external loads can cause changes in the stress distribution, leading to warping, dimensional deviations, and even crack initiation, thereby reducing fatigue life [1]. Therefore, accurately obtaining the RSF is crucial for controlling the residual stress and ensuring the reliability of structural components.

The current methods for obtaining residual stress primarily include residual stress prediction and measurement methods. The prediction methods involve simulating the manufacturing process of the blanks using numerical simulation to obtain the RSF inside the material. Ortmann-Ishkina et al. [2] constructed a two-dimensional simulation model of the die-forging process using finite element software to investigate the residual-stress distribution law of E355 steel pipes. Bouissa et al. [3] used a three-dimensional finite element model to simulate high-strength steel forgings and investigated the deformation and residual stresses induced by water quenching. Wang et al. [4] constructed a three-dimensional ultrasonic rolling model of Ti-6Al-4 V alloy plates and verified the reliability of the model by analyzing the residual stress results. Gong et al. [5] used FEM to simulate the quenching process of 7050 aluminum alloy die-forging parts and analyzed the difference between the RSF obtained by simulation and the results obtained by the layer-by-layer and X-ray diffraction methods. Ren et al. [6] studied the distribution patterns of the RSF in 7055 aluminum alloy flat blanks of different widths using FEM. Owing to factors such as material heterogeneity and complex boundary conditions, accurate modeling is challenging. Residual stress prediction methods can provide accurate distribution trends of RSF in die-forging structural parts; however, relying on distribution trends alone is insufficient to guarantee the reliability of structural components.

The measurement methods involve calculating the internal residual stress of a material by measuring stress-related physical quantities and using the relationship between these quantities and the residual stress. Depending on whether or not the measurement process causes damage or destruction to the blank, the RSF measurement methods can be divided into destructive and nondestructive methods. Destructive measurement methods use physical or chemical means to damage a material, causing a local imbalance in the RSF. The internal residual stress of a material can be estimated by measuring the strain or displacement caused by an imbalance. Destructive measurement methods include the hole drilling [7, 8], layer removal [9], compliance [10, 11] and contour methods [12]. Nondestructive measurement methods calculate the magnitude of the residual stresses from the propagation speed or radiation within the local material. Nondestructive methods for measuring RSFs include ultrasonic measurements [13], X-ray diffraction [14], neutron diffraction [15], and gamma-ray diffraction [16]. These methods calculate residual stress by measuring certain inherent properties within a specific material region [17]. Most traditional RSF measurement methods are strain based and can only obtain the local or segmented residual stress, making it difficult to obtain the complex global RSF of parts. In addition, these methods are inefficient and cannot fulfill the demands of machining deformation control for die-forging structural parts with global and uneven RSF.

With the rapid development of sensors and information technology in recent years, more data from machining processes have become available for monitoring. This has led to new methods being proposed for inferring RSF using process monitoring data. The key idea behind these methods is to establish a relationship between the global RSF and macro-level monitoring data, enabling the inference of internal residual stresses based on the monitored data from the machining process. Initially, these methods were developed to address the issue of inferring the RSF in prestretched plate blanks. Wang et al. [18] proposed a method that combines data and mechanisms to infer the initial residual stress. This method involves creating a mechanistic model using finite element software to link force monitoring data with the RSF; the initial RSF of prestretched plate blanks is then iteratively inferred through a strategy gradient method. Within a Bayesian framework, Hu et al. [19] treated the RSF as a latent variable and used force monitoring data from the machining process as observations to perform Bayesian measurement, achieving accurate predictions of the RSF in prestretched plate blanks. Zhao et al. [20] developed a method based on deformation-force monitoring data to infer the RSF. They defined the concept of the deformation force and established a general formula for relating it to the RSF distribution in blanks using the principle of virtual work, allowing for global RSF inference.

The deformation-force-based method shows great potential in addressing the challenge of inferring the RSF in die-forged parts. However, for die-forging parts with complex RSF distributions and irregular geometries, a large number of parameters are required for solving these problems; therefore, it is difficult to address the inverse problem because of the ill-conditioned problem caused by a large number of parameters. Introduction of priors is an effective method for solving inverse problems. Therefore, representing the residual stress with a certain number of parameters according to prior knowledge and using deformation force monitoring data to solve these parameters is key to inferring the residual stress in die-forging parts.

This paper presents a global RSF inference method for die-forging structural parts based on the fusion of monitoring data and distribution priors, where prior knowledge represents the distribution trend of the RSF obtained by simulating the forging and heat treatment processes of die-forging structural parts. Using the distribution trends to divide the RSF region, a low-dimensional characterization of the RSF can be achieved, which reduces the number of parameters required to solve it. Within the Bayesian framework, the deformation force monitoring data were used to infer the RSF of the die-forging structural parts. Finally, the effectiveness of this method was validated in both simulation and actual environments.

## Methods

### Overview of global RSF inference method

**Fig. 1:**
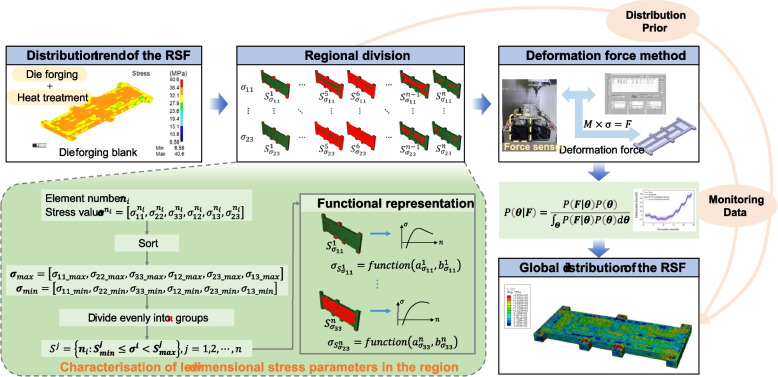
Overview of the method

In this study, a global RSF inference method for die-forging structural parts based on the fusion of monitoring data and distribution priors is proposed. First, a numerical simulation model of the manufacturing process of die-forging parts was established to obtain the distribution trends of the RSF within the blank. Using the distribution trends as a prior, the RSF of the die-forging parts can be characterized in low dimensions. Within the Bayesian framework, the RSF characterization parameters of the die-forging parts were inferred. An overview of the method is depicted in Fig. 1.

### Prior acquisition of RSF distribution in die-forging parts

The prior distribution of RSFs in die-forging parts can be obtained by simulating the die forging and heat treatment processes using numerical simulation methods. Relevant studies indicate that these processes are the main factors that determine the internal RSF of die-forging parts [21, 22].

First, the die-forging stage was implemented using finite element analysis software, where the blanks, dies, material property parameters, and forging process parameters were set. During the forging of the blank, the geometric shape and microstructure of the metal underwent significant changes owing to the applied forging machinery.

Subsequently, a heat treatment process was applied to the forging blank to further enhance its mechanical properties, which was simulated at a predefined temperature. The residual stresses within the material were obtained after the simulation.

It is believed that under the assurance of the FEM, the simulated distribution trends of the RSF throughout the entire component will show good consistency with the actual results. By contrast, the difference between the simulation and actual results mainly reflects the numerical differences in the RSF. The RSF distribution trends obtained from the numerical simulations are of considerable importance for the subsequent characterization and inference of the RSF.

### Characterization and inference of RSF in die-forging parts

During machining of die-forging parts, the equilibrium of the RSF cannot be maintained. The unbalanced stress acts on the clamping fixture in the form of a force that resists deformation, which is called the deformation force and it reflects the global RSF action on the deformation. Therefore, a deformation-force-based global RSF inference method was proposed [20]. However, a key problem that remains to be addressed is that the RSF-solving process of RSF needs a limited number of unknown parameters representing the RSF. Therefore, the characterization of the complex RSF in the forged parts is fundamental to solving the RSF based on the deformation force.

#### RSF characterization method for die-forging parts

Although the distribution of RSF in die-forging structural parts is complex, many element stress values are similar. Therefore, grouping elements with similar stress levels can reduce the complexity of the RSF characterization. This study proposes grouping elements within a certain stress level range based on the RSF distribution trends obtained from numerical simulations. A function represents the stress distribution within each group, ultimately achieving a low-dimensional characterization of the RSF in die-forging structural parts through a limited number of function parameters.

In the numerical simulation method, the global RSF of die-forged parts is divided into ${n}_{i}$ elements, and the stress value ${{\varvec{\sigma}}}^{{n}_{i}}$ in each element is:

1 $${{\varvec{\sigma}}}^{{n}_{i}}=\left[{\sigma }_{11}^{{n}_{i}},{\sigma }_{22}^{{n}_{i}},{\sigma }_{33}^{{n}_{i}},{\sigma }_{12}^{{n}_{i}},{\sigma }_{13}^{{n}_{i}},{\sigma }_{23}^{{n}_{i}}\right]$$

After obtaining each element’s identifier and corresponding stress value, the elements within the same stress level range can be grouped according to certain rules. The uniform division method defined a range of similar stresses to ensure that the stress levels within each group were similar. Taking the $x$-direction stress ${\sigma }_{11}$ in the element as an example, the maximum value ${\sigma }_{11\_max}$ and minimum value ${\sigma }_{11\_min}$ stress among all elements are determined first. Subsequently, the number of groups $n$ was determined based on the amount of deformation force data obtained during machining. The range between the maximum value ${\sigma }_{11\_max}$ and minimum value ${\sigma }_{11\_min}$ is divided into $n$ equal parts. Each group ${S}_{11}^{j},\text{where }j=\text{1,2},\cdots ,n,$ is defined by a stress range as:

2 $${S}_{11}^{j}=\left\{{n}_{i}:{S}_{11\_min}^{j}\le {\sigma }_{11}^{{n}_{i}}<{S}_{11\_max}^{j}\right\};j=\text{1,2},\cdots ,n$$

where ${n}_{i}$ is the element number and $n$ is the number of groups.

Next, the RSF within each group was characterized. If the stress values of all elements within a group are approximated to the same value, the number of errors will increase. Therefore, an attempt is made to fit the stress distribution within each group using a function. The stress distribution within group ${S}_{11}^{j}$ is expressed as:

3 $${\sigma }_{11}^{j}=function\left({n}_{k}\right);{n}_{k}\in {S}^{j}$$

The number of parameters in the function is the number of parameters required to represent an RSF. A further reduction in the number of parameters within the function is critical. Because the RSF inside a part does not change abruptly in theory, the stress values of each element within a group can be fitted using a simple linear function after being arranged in ascending order. This is expressed as:

4 $${\sigma }_{11}^{j}=function\left({a}_{11}^{j},{b}_{11}^{j}\right)=\left[{a}_{11}^{j}{{\varvec{n}}}^{{\varvec{j}}}+{b}_{11}^{j}\right];j=\text{1,2},\cdots ,n$$

where ${{\varvec{n}}}^{j}$ is a column vector comprising the number of all elements in group ${S}^{j}$ sorted in ascending order. This reduces the number of parameters in a single direction within group ${S}^{j}$ to two, namely, ${a}_{11}^{j}$ and ${b}_{11}^{j}$. The residual stress in other directions within the elements can also be characterized using the above method. The algorithmic process is as follows.

**Figure:**
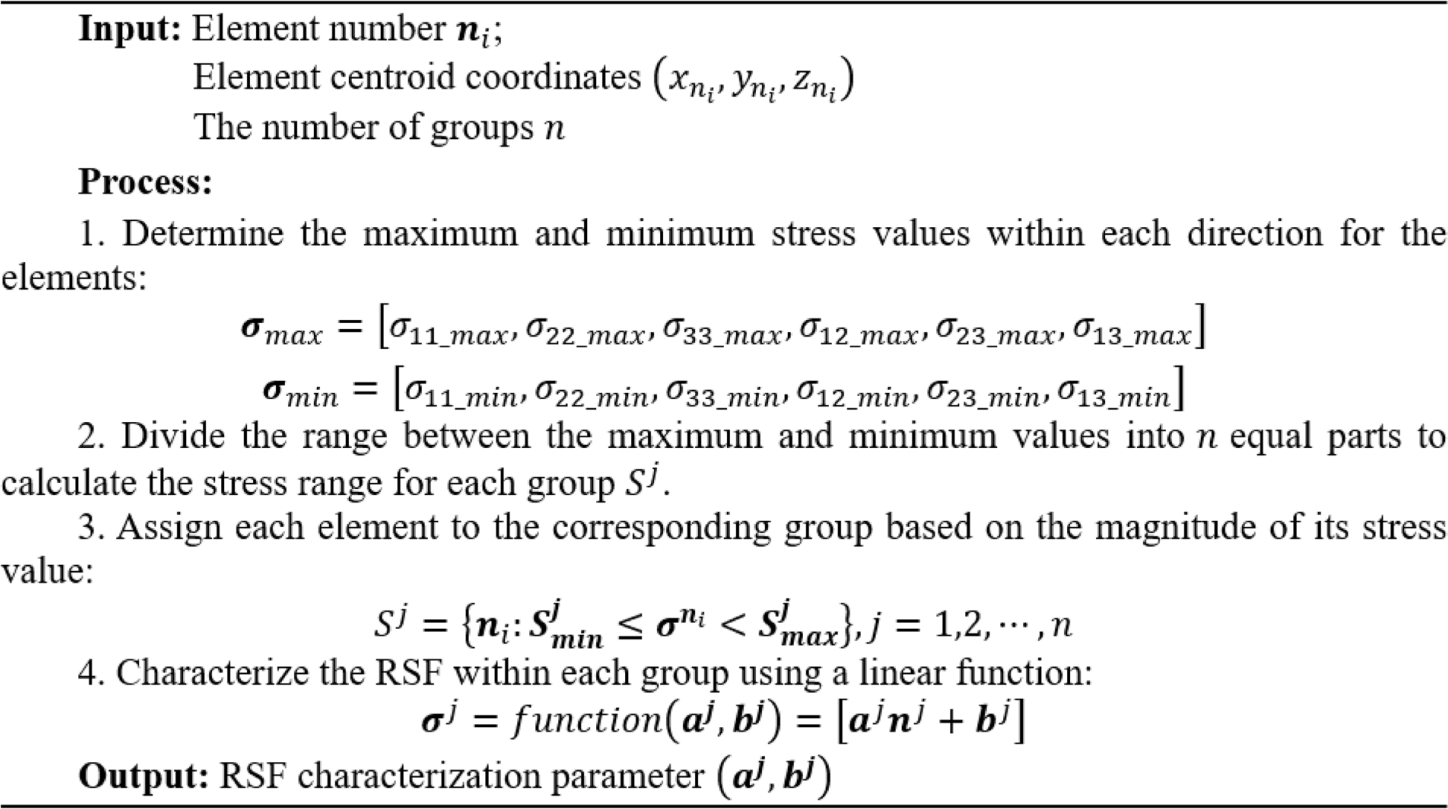
**Algorithm 1** Low-dimensional characterization method of RSF

Ultimately, using the method proposed in this study, the RSF within the die-forging parts can be characterized by the following equation:

5 $${{\varvec{\sigma}}}_{0}=\left[\begin{array}{c}\begin{array}{c}\begin{array}{c}\begin{array}{c}{a}_{11}^{1}{{\varvec{n}}}^{1}+{b}_{11}^{1}\\ {a}_{11}^{2}{{\varvec{n}}}^{2}+{b}_{11}^{2}\\ \vdots \end{array}\\ {a}_{11}^{n}{{\varvec{n}}}^{n}+{b}_{11}^{n}\end{array}\\ \begin{array}{c}\begin{array}{c}{a}_{22}^{1}{{\varvec{n}}}^{1}+{b}_{22}^{1}\\ {a}_{22}^{2}{{\varvec{n}}}^{2}+{b}_{22}^{2}\\ \vdots \end{array}\\ {a}_{22}^{n}{{\varvec{n}}}^{n}+{b}_{22}^{n}\end{array}\end{array}\\ \begin{array}{c}\vdots \\ {a}_{23}^{n}{{\varvec{n}}}^{n}+{b}_{23}^{n}\end{array}\end{array}\right]$$

By introducing the trends of the RSF distribution under numerical simulation, the number of parameters required to characterize the RSF in die-forging parts is reduced to $2\times n\times 6$, where 6 denotes the six-directional residual stresses inside the material. This method significantly reduces the number of parameters required for characterization while maintaining the ability to accurately characterize complex RSF.

#### Global RSF inference method for die-forging parts based on deformation force

With the low-dimensional characterization method, the inference of RSFs can be achieved based on the deformation-force-based method. The relationship between the deformation force during machining and the initial RSF inside the material is derived by combining the principle of virtual work.

6 $${\varvec{M}}{{\varvec{\sigma}}}_{0}={\varvec{F}}$$

where ${\varvec{M}}$ is the volume coefficient matrix of the part, ${{\varvec{\sigma}}}_{0}$ is the initial RSF within the material, and ${\varvec{F}}$ is the deformation force data monitored at the clamping points during the machining process.

By incorporating the proposed RSF characterization method (Eq. 4) into Eq. 5, the following equation was obtained:

7 $${\varvec{M}}\left[\begin{array}{c}\begin{array}{c}\begin{array}{c}{a}_{11}^{1}{{\varvec{n}}}^{1}+{b}_{11}^{1}\\ {a}_{11}^{2}{{\varvec{n}}}^{2}+{b}_{11}^{2}\\ \vdots \end{array}\\ {a}_{11}^{n}{{\varvec{n}}}^{n}+{b}_{11}^{n}\end{array}\\ \vdots \\ {a}_{23}^{n}{{\varvec{n}}}^{n}+{b}_{23}^{n}\end{array}\right]={\varvec{F}}$$

Hence, the problem of inferring the RSF in the forged parts can be transformed into a problem of inferring the function parameters. In an actual environment, deformation force data are often affected by various uncertainties such as machine noise and vibrations during the machining process. These uncertainties can lead to instability when solving the RSF because the problem is inherently ill-posed. In particular, small variations or noise in the observed data can result in significant changes in the solution, rendering the inference of the RSF inaccurate.

To address this issue, Bayesian inference was employed to determine the RSF in forged parts. This approach is well-suited for solving inverse problems under uncertainty. Within the Bayesian framework, the RSF is considered a random and unobservable field described by a prior distribution. The posterior distribution was refined by incorporating prior knowledge and continuously updating it with the observed deformation force data. This refinement leads to a more stable and reliable estimate of the RSF. This approach effectively mitigated the impact of noise and other uncertainties in the data. The prior assumptions were then continuously updated using the observed data, and the posterior probabilities were calculated.

The problem of inferring the RSF in die-forging parts can be expressed by the following equation:

8 $$P\left({\varvec{\theta}}|{\varvec{F}}\right)=\frac{P\left({\varvec{F}}|{\varvec{\theta}}\right)P\left({\varvec{\theta}}\right)}{{\int }_{\boldsymbol{\Theta }}P\left({\varvec{F}}|{\varvec{\theta}}\right)P\left({\varvec{\theta}}\right)d{\varvec{\theta}}}$$

9 $${\varvec{\theta}}=\left\{{a}_{11}^{1},{a}_{11}^{2},\cdots ,{a}_{11}^{n},{a}_{22}^{1},\cdots {a}_{23}^{n},{b}_{11}^{1},{b}_{11}^{2},\cdots ,{b}_{11}^{n},{b}_{22}^{1},\cdots {b}_{23}^{n}\right\}$$

where ${\varvec{F}}$ is the deformation force monitoring data during the machining process, $\boldsymbol{\Theta }$ is the space composed of all possible values of the parameter ${\varvec{\theta}}$, ${\varvec{\theta}}$ are the function parameters representing the RSF, $P\left({\varvec{\theta}}|{\varvec{F}}\right)$ is the posterior probability of the parameter ${\varvec{\theta}}$, $P\left({\varvec{\theta}}|{\varvec{F}}\right)$ is the likelihood probability, and $P\left({\varvec{\theta}}\right)$ is the prior probability that provides the estimation of the probability of the parameter ${\varvec{\theta}}$ based on prior information. Under this formulation, the problem of inferring the RSF in die-forging parts is transformed into continuously updating the prior assumptions through deformation force observational data and calculating the posterior probability $P\left({\varvec{\theta}}|{\varvec{F}}\right)$.

## Results and Discussion

To verify the validity of the proposed methodology for inferring the RSF in die-forging parts, the residual-stress distribution was obtained as a prior in the finite element software, and verification was conducted in both theoretical and practical experimental environments.

### Prior acquisition of RSF distribution

**Fig. 2:**
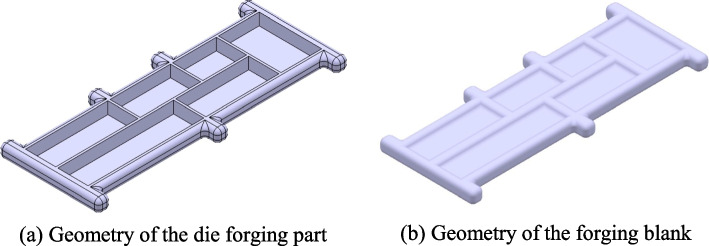
Geometry of the die-forging part

To obtain the prior distribution of the RSF in the die-forging parts, the DEFORM finite element analysis software was used to simulate the thermal forging and heat treatment processes. A die-forging part with typical pocket features of aircraft structural parts was designed. The size of the part was 600 mm × 180 mm × 30 mm, and it was made of 7050 aluminum alloy. The geometry of this part is shown in Fig. 2(a) and contains six pocket features on the upper surface. The forging blank is shown in Fig. 2(b), where the depth of the forging pocket features was 10 mm.

The material properties of the 7050 aluminum alloy were set according to the relevant literature [23], as listed in Table 1. The Arrhenius constitutive model is one of the most widely used models for effectively describing the rheological behavior of materials at high temperatures [24].

**Table 1:** 7050 aluminum alloy material parameter setting

Parameter	Value
Arrhenius constitutive model	$\dot{\overline{\varepsilon }}=A{\left[sinh\left(\alpha\overline{\sigma }\right)\right]}^{n}exp\left[-\Delta H/\left(R{T}_{abs}\right)\right]$
Structure factor A	3.7050E6
Constant $\alpha$	0.01142
Activation energy $\Delta H$	98,693.19 $J/mol$
Hardening index $n$	7.3658
Gas constant $R$	8.3145
Yield criterion	Von Mises
Hardening rule	Isotropic
Young modulus	68,900 $N/{mm}^{2}$
Poisson’s ratio	0.33
Thermal expansion coefficient	2.3e-05 $/^\circ{\rm C}$
Pyroconductivity	138 $W/\left(m\cdot K\right)$
Radiation ratio	0.7
Specific heat capacity	3.04508 $N/{mm}^{2}\cdot^\circ{\rm C}$

In the heat treatment process, the relevant parameters were set according to the literature [25], as listed in Table 2. Under this setting, the heat treatment schedule for the forging blank involved solution treatment followed by quenching. The solution treatment temperature was set to 470°C, and the quenching medium was water at 20°C.

**Table 2:** Heat treatment parameter setting

Parameter	Value
Solution treatment temperature	420℃
Quenching medium	380℃
Quenching temperature	1 mm/s

**Fig. 3:**
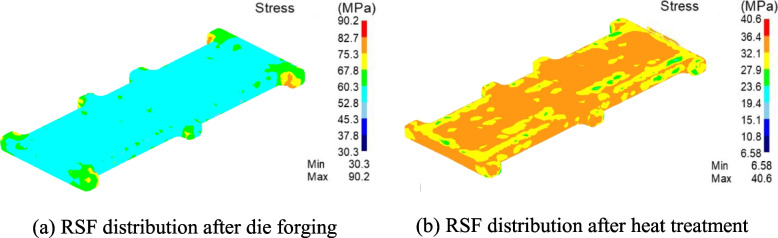
RSF at the end of forging and heat treatment

An RSF distribution map of the blank at the end of the forging process is shown in Fig. 3(a). It can be observed that the minimum equivalent stress is 30.3 MPa, and the maximum equivalent stress is 90.2 MPa. At the same time, the fact that the blank was still at a relatively high temperature allowed the molecules within the material to exhibit high mobility, resulting in a relatively uniform global equivalent stress. A cloud diagram of the RSF distribution map of the blank after the heat treatment process was completed is shown in Fig. 3(b). It can be observed that with the heat treatment process, the distribution of the RSF within the blank was non-uniform.

### Theoretical environment verification

In the simulation environment, the RSF distribution of the die-forging parts obtained from the DEFORM finite element simulation was considered as a prior, thereby conducting a low-dimensional characterization of the RSF. Simultaneously, the machining process of the part was simulated to obtain the deformation force data in the theoretical environment. Subsequently, the inferred function parameters representing the RSF were compared with the real values to verify the validity of the method.

**Fig. 4:**
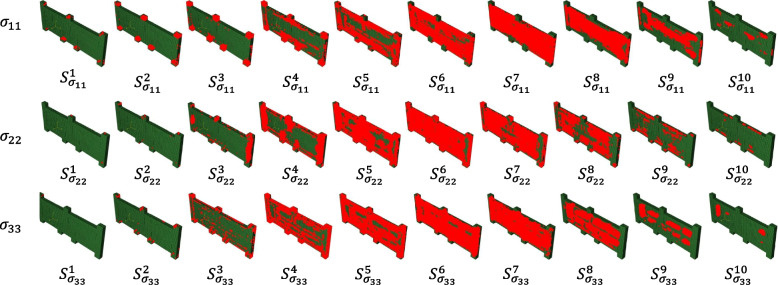
RSF partitioning result diagram

First, each element and the corresponding stress values were exported to the DEFORM software. The exported data shows that the magnitude of shear stresses ${{\varvec{\sigma}}}_{12}, {{\varvec{\sigma}}}_{13}\text{ and }{{\varvec{\sigma}}}_{23}$ is approximately 8% of the principal stresses ${{\varvec{\sigma}}}_{11}, {{\varvec{\sigma}}}_{22}\text{ and }{{\varvec{\sigma}}}_{33}$. The principal stresses have a significantly greater influence on machining deformation than shear stresses, which have negligible effects. Consequently, when addressing machining deformation, the focus should be on the principal stresses, whereas the shear stresses can be disregarded to simplify the analysis without significantly affecting the accuracy of the results. In addition, elements within a certain stress-level range were aggregated into one group by considering the model accuracy and computational efficiency. In this case, the number of groups n was set to ten to avoid large differences in stress levels within the groups. The maximum stress difference between the elements in each group was 20 MPa. Through the RSF characterization method proposed in this paper, the RSF in three directions of the die-forging parts, ${{\varvec{\sigma}}}_{11}, {{\varvec{\sigma}}}_{22}\text{ and }{{\varvec{\sigma}}}_{33}$, are divided into 10 groups, respectively, as shown in Fig. 4. The total number of parameters to be solved is 60.

**Fig. 5:**
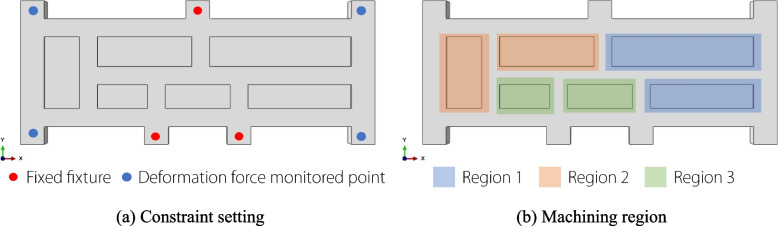
Simulation environment setup

The ABAQUS software was used to apply the RSF of the forged structure to obtain the deformation force monitoring data of the part in the simulation environment. The “birth and death element method” was used to simulate the machining process of the part. The clamping constraints of the part during the simulation were set as shown in Fig. 5(a). The clamping form refers to the 6 + X positioning method [26], where fixed clamping points are used to locate the six degrees of freedom of the part during the machining process. Four deformation force monitoring points were simulated using spring constraints that were designed with extremely high stiffness to constrain the deformation of the part. In the simulation, the spring stiffness was set to 500,000 N/mm.

The machining of a part follows the layer-priority principle. The removed material was divided into eight layers at a cutting depth of 2 mm, and each layer was divided the six pockets into three regions, as shown in Fig. 5(b). Deformation force data were recorded when the material was removed from one region, resulting in a total number of 96 deformation force data points.

**Fig. 6:**
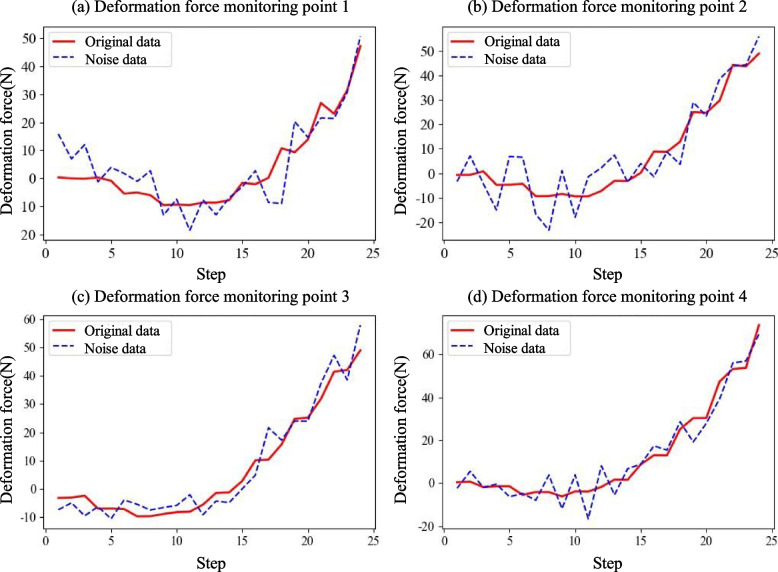
Comparison of deformation force data with original data and noise data

To simulate the problem of RSF prediction in real scenarios, Gaussian noise with a mean of 0 and standard deviation of 5 N was added to the simulated deformation force data, as shown in Fig. 6.

The 60 parameters to be inferred were divided into two groups, ${{\varvec{a}}}^{j}$ and ${{\varvec{b}}}^{j}$, using the PyMC3 function library in Python, and two multivariate Gaussian distributions are sampled as priors for latent variables. Each multivariate Gaussian distribution contains a mean function and covariance function as follows:

10 $${{\varvec{a}}}^{j}\sim MGD\left({mu}_{a},{cov}_{a}\right)$$

11 $${{\varvec{b}}}^{j}\sim MGD\left({mu}_{b},{cov}_{b}\right)$$

where ${mu}_{a}$ and ${mu}_{b}$ are 1 × 30 vectors that represent the mean prior to each parameter to be inferred. The covariance functions, ${cov}_{a}$ and ${cov}_{b}$ were set as a 30 × 30 identity matrix. The observed deformation force data were assumed to follow a normal distribution as follows:

12 $${\varvec{F}}\sim MGD\left({mu}_{F},{sigma}_{F}\right)$$

where ${mu}_{F}$ is a 1 × 96 vector representing the deformation force data and ${sigma}_{F}$ represents the measurement noise in the deformation force caused by various uncertainties, which is set to a standard deviation of 1 N.

Combined with Eq. 6, the mechanical relationship between the RSF and the deformation force data within the Bayesian framework is as follows:

13 $${\varvec{F}}\sim {\varvec{M}}\times MGD\left({mu}_{a},{cov}_{a}\right)\times {\varvec{n}}+{\varvec{M}}\times MGD\left({mu}_{b},{cov}_{b}\right)$$

**Fig. 7:**
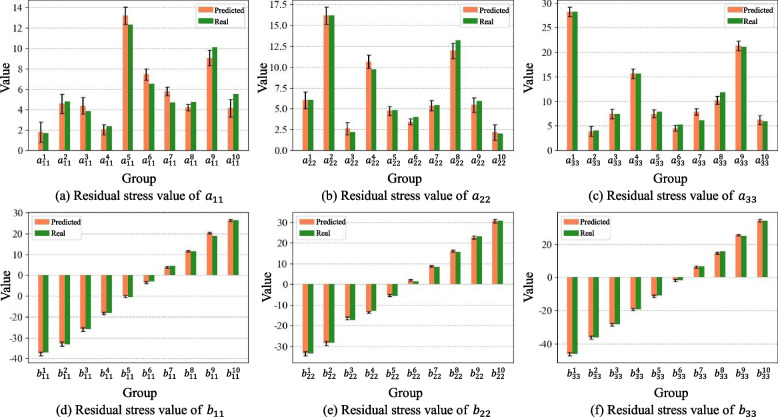
Results of the RSF characterization parameters under noisy data

Finally, the posterior distributions of the inferred parameters were sampled using the Markov chain MCMC method. Using noisy deformation force data, the posterior distribution of each parameter was obtained after 2000 iterations, and the results are shown in Fig. 7. The calculated average absolute error of parameter **a** was 0.54, with an average relative error of 9.14%. The average absolute error of parameter **b** is 0.382, with an average relative error of 1.51%.

**Fig. 8:**
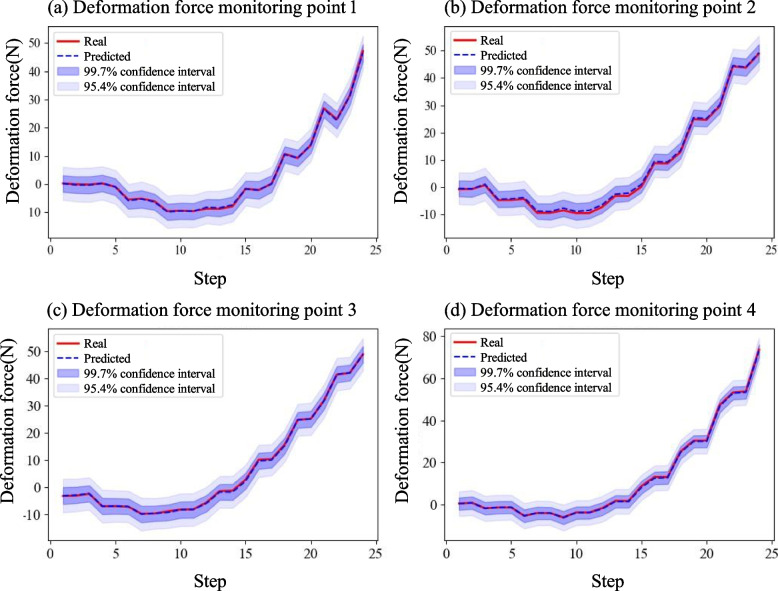
Posterior distribution of deformation force under noisy data

In addition, the posterior distribution of the deformation force can be obtained by inference, as shown in Fig. 8. The average absolute error of the deformation force prediction was 0.294 N.

The above results indicate that the proposed method can accurately infer the RSF of die-forging parts and verify its effectiveness.

### Actual machining verification

**Fig. 9:**
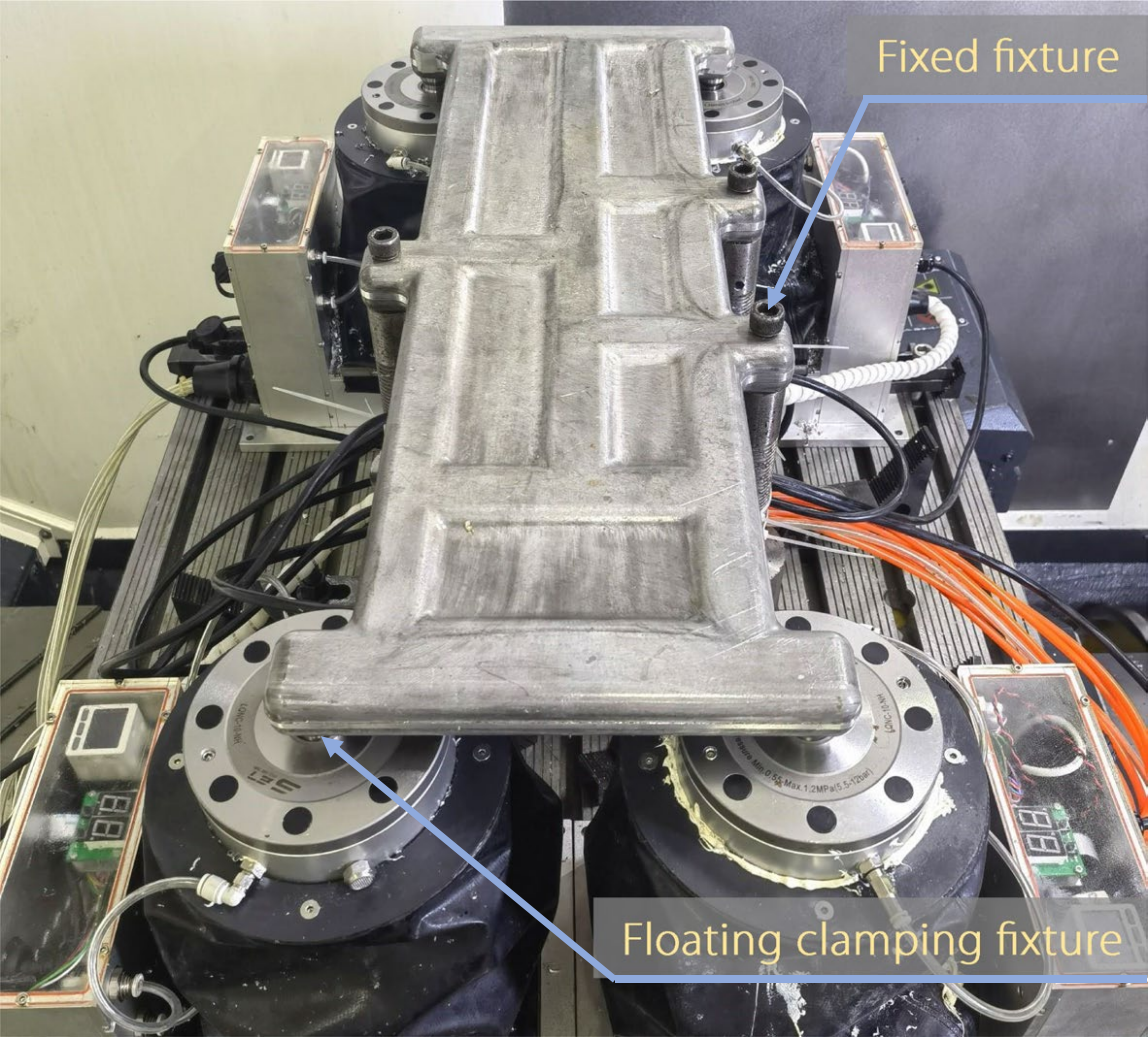
Clamping setup for the actual machining environment

Actual machining experiments on the die-forging parts were conducted at the DMU 80P five-axis machining center, as shown in Fig. 9. The blank size and forming process of the die-forged structure were consistent with the simulation parameters. The cutting parameters used during machining are listed in Table 3. The machining process and parameters were consistent with those used in the simulation environment. The deformation force data monitored by intelligent fixtures installed with force sensors during the machining process are listed in Table 4. There was a significant difference between the deformation force data monitored during the actual machining process and the deformation force data from the simulation environment. This suggests a disparity in the RSF distribution between the simulation environment and actual part.

**Table 3:** Machining parameter

Spindle speed	Cut width	Feed speed	Cutting depth	Tool diameter
6000 turn/min	10 mm	1200 mm/min	2mm	20 mm

**Table 4:** Deformation force monitoring data (N)

Level number	Region number	Point 1	Point 2	Point 3	Point 4
Level 1	Region1	−74	−73	−110	−150
	Region2	−83	−63	−112	−160
	Region3	−231	−133	−261	−262
Level 2	Region1	−323	−200	−397	−420
	Region2	−307	−204	−407	−445
	Region3	−491	−293	−568	−582
Level 3	Region1	−563	−359	−669	−715
	Region2	−555	−368	−689	−735
	Region3	−744	−450	−831	−846
Level 4	Region1	−790	−522	−912	−938
	Region2	−798	−520	−927	−948
	Region3	−963	−585	−1037	−1031
Level 5	Region1	−983	−650	−1083	−1073
	Region2	−997	−648	−1098	−1083
	Region3	−1107	−693	−1162	−1131
Level 6	Region1	−1115	−700	−1171	−1127
	Region2	−1128	−702	−1190	−1138
	Region3	−1170	−715	−1201	−1141
Level 7	Region1	−1150	−680	−1172	−1089
	Region2	−1134	−677	−1136	−1070
	Region3	−1139	−651	−1136	−1043
Level 8	Region1	−1059	−588	−1061	−928
	Region2	−1052	−580	−1049	−913
	Region3	−944	−504	−954	−814

Because the real RSF within the material is unknown in the actual machining environment, the validation approach for the environment is as follows. The RSF within the part was inferred using the deformation force data obtained during the first seven layers of CNC machining. Subsequently, the deformation force data in the eighth layer of the machining process were predicted based on the inferred RSF and compared with the actual machining deformation force data to verify the effectiveness of the proposed method in an actual machining environment.

**Fig. 10:**
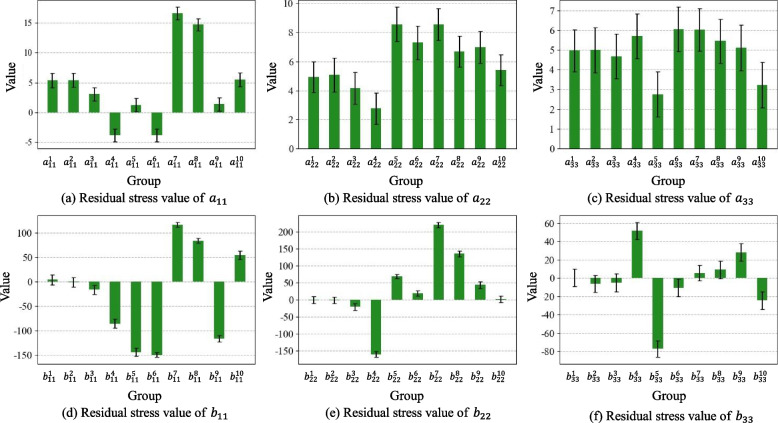
Results of the RSF characterization parameters in the actual machining environment

In the RSF inference process, except for the size of the deformation force monitoring data ${mu}_{F}$, which was reduced from 1 × 96 to 1 × 84, the remaining settings were the same as those in the simulation environment. After 2000 iterations, the posterior distribution of the characterization parameters was obtained, as shown in Fig. 10.

**Fig. 11:**
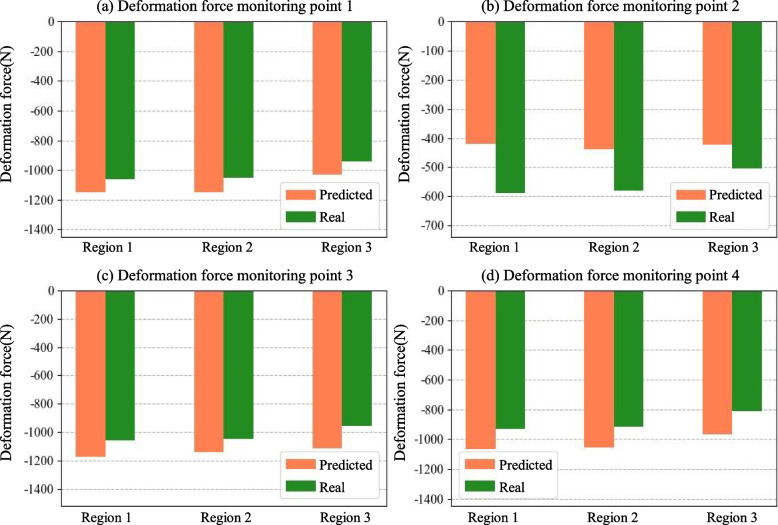
Comparison of the deformation force of the eighth layer in the actual machining environment

The deformation force data during machining of the eighth layer of the part were predicted using the inferred RSF, and the prediction results are shown in Fig. 11. The predicted results indicated that the average absolute error of the deformation force during machining of the eighth layer was 121.4 N, with an average relative error of 15.1%.

**Fig. 12:**
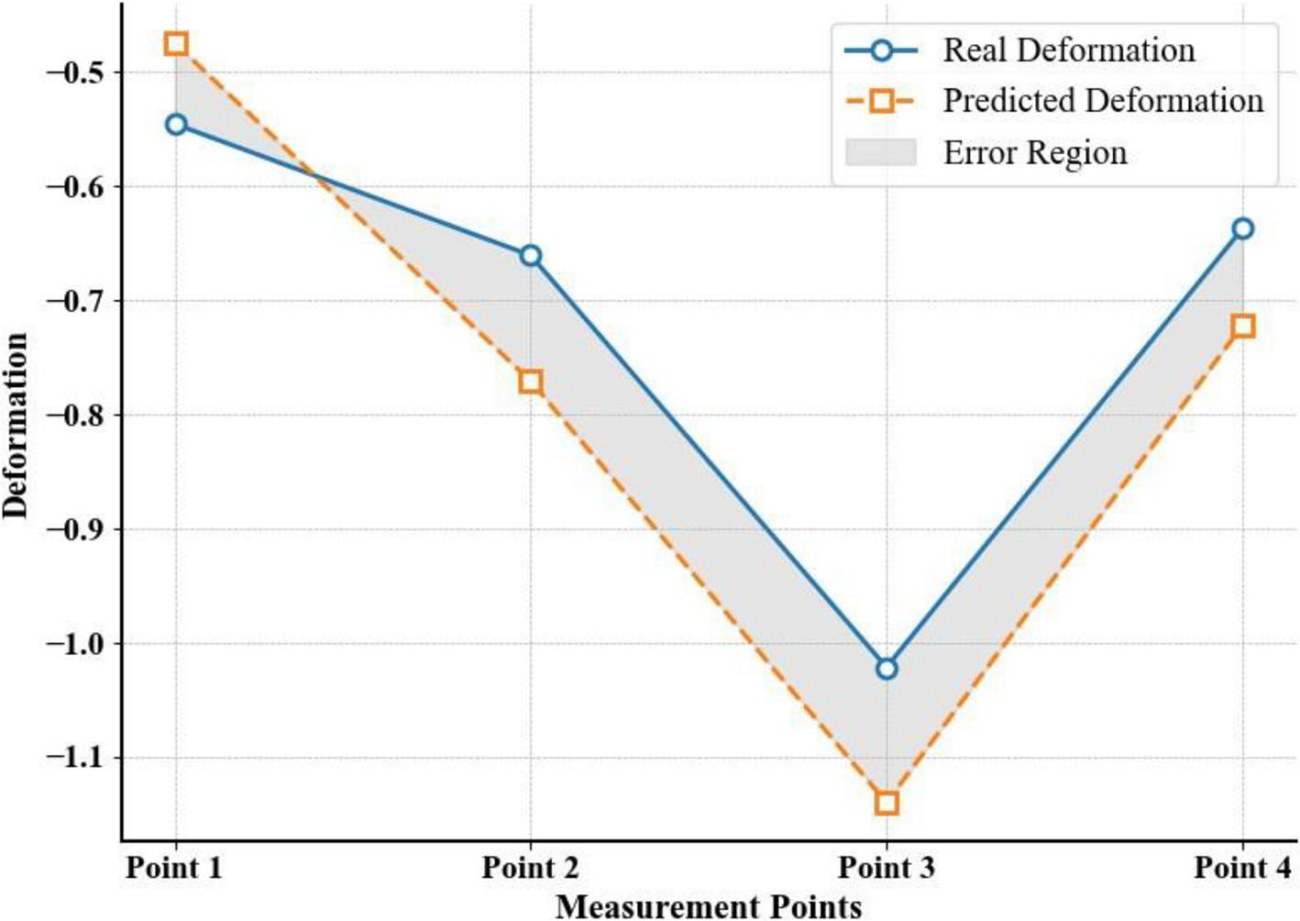
Comparison of real and predicted deformation

A deformation force was applied as an external load to the final part to explore the impact of the RSF inference error on subsequent deformation predictions. A comparison of the deformation of the part under both real and predicted deformation forces is shown in Fig. 12. The mean absolute error at each node was 0.0962 mm. By comparing the deformation of the part along the thickness direction at all the nodes, it was calculated that the error introduced by the RSF inference led to an error of approximately 13.7% in subsequent deformation predictions. The discrepancies between the actual and predicted deformation values can be attributed to two primary factors: (1) potential errors in the RSF inference, and (2) measurement errors of the part deformation. Despite these discrepancies, the average absolute error of the four corner points is less than 0.1 mm, indicating that the proposed method still offers reliable predictions for machining deformation, even in the presence of some variation in the RSF distribution. This indicates that the method can infer the RSF with a reasonable degree of accuracy, thereby fulfilling the requirements of conventional machining deformation prediction.

## Conclusions

The RSF affects the entire lifecycle of structural parts. Global inference of the RSF can provide a basis for process control. The RSF distribution of die-forging parts is complex; therefore, it is difficult to characterize. This study proposes a method for global RSF inference in die-forging structural parts based on the fusion of monitoring data and distribution priors. First, the distribution trend of the RSF within the blank of die-forging parts was obtained through numerical simulations of the die forging and heat treatment processes. Based on this, the distribution trend is utilized as a prior partition for the low-dimensional characterization of the RSF inside the die-forging parts. The RSF characterization parameters of the die-forging component were inferred from the deformation force monitoring data collected during the machining process using a Bayesian framework. The proposed global RSF inference method achieved a stable and accurate inference of the RSF for die-forging structural parts.

Although this study focused on aluminum alloy components, the proposed method is generic and applicable to other materials. Specifically, it can be extended to titanium alloys and other linear elastic materials, where a low-dimensional characterization of the RSF can be obtained by leveraging prior knowledge of the residual-stress distribution trend. The method can be used to infer the RSF from monitoring data. The flexibility of the method makes it suitable for a wide range of industrial applications, including the aerospace, automotive, and manufacturing industries, where residual stress analysis is critical.

However, some areas require further improvement. From the experimental data analysis, two main factors affected the accuracy of the global RSF inference. The first was the difference between the theoretical and actual RSF distributions. The second is the number and division strategy of the RSF groups as well as the use of different functions to characterize the stress in the group. In future research, the prior acquisition of the RSF distribution trend of the die-forging parts, in addition to the FEM, will require many residual stress measurement methods, such as the drilling and ultrasonic measurement methods, which can be combined with the prior information obtained by multiple methods to obtain more accurate residual stress solutions. In addition, other partitioning strategies and characterization functions may improve the accuracy of the global RSF inference.

## Data Availability

The datasets used and/or analyzed in the current study are available from the corresponding author upon reasonable request.

## Abbreviations

RSF: Residual stress field
FEM: Finite element method
MAE: Mean absolute error

## References

[1] Zhao YJ, Liu CQ, Zhao ZW, Tang K, He D (2022) Reinforcement learning method for machining deformation control based on meta-invariant feature space. Vis Comput Ind Biomed Art 5(1):27. 10.1186/s42492-022-00123-2 36418749 10.1186/s42492-022-00123-2 PMC9684396

[2] Ortmann-Ishkina S, Charni D, Herrmann M, Liu Y, Epp J, Schenck C et al (2021) Development of residual stresses by infeed rotary swaging of steel tubes. Arch Appl Mech 91(8):3637–3647. 10.1007/s00419-021-01905-5

[3] Bouissa Y, Bohlooli N, Shahriari D, Champliaud H, Morin JB, Jahazi M (2020) FEM modeling and experimental validation of quench-induced distortions of large size steel forgings. J Manuf Process 58:592–605. 10.1016/j.jmapro.2020.08.042

[4] Wang F, Men X, Liu YJ, Fu XL (2020) Experiment and simulation study on influence of ultrasonic rolling parameters on residual stress of Ti-6Al-4V alloy. Simul Model Pract Theory 104:102121. 10.1016/j.simpat.2020.102121

[5] Gong H, Wu YX, Zhang T, Liu YQ, Li C, Ji H et al (2017) Quenching residual stresses in T-section 7050 aluminum alloy forging. Mechanika 23(3):353–358. 10.5755/j01.mech.23.3.14259

[6] Ren WC, Li YN, Zhang YA, Tong YZ, Li XW, Li ZH et al (2022) Prediction of residual stress field on the surface of quenched 7055 aluminium alloy plates. Mater Res Express 9(3):036502. 10.1088/2053-1591/ac57d9

[7] Mathar J (1934) Determination of initial stresses by measuring the deformations around drilled holes. Trans ASME 56(3):249–254. 10.1115/1.4019712

[8] Treuting RG, Read WT (1951) A mechanical determination of biaxial residual stress in sheet materials. J Appl Phys 22(2):130–134. 10.1063/1.1699913

[9] Virkar AV (1990) Determination of residual stress profile using a strain gage technique. J Am Ceram Soc 73(7):2100–2102. 10.1111/j.1151-2916.1990.tb05276.x

[10] Vaidyanathan S, Finnie I (1971) Determination of residual stresses from stress intensity factor measurements. J Basic Eng 93(2):242–246. 10.1115/1.3425220

[11] Prime MB, Hill MR (2002) Residual stress, stress relief, and inhomogeneity in aluminum plate. Scr Mater 46(1):77–82

[12] Prime MB (2001) Cross-sectional mapping of residual stresses by measuring the surface contour after a cut. J Eng Mater Technol 123(2):162–168. 10.1115/1.1345526

[13] Javadi Y, Akhlaghi M, Najafabadi MA (2013) Using finite element and ultrasonic method to evaluate welding longitudinal residual stress through the thickness in austenitic stainless steel plates. Mater Des 45:628–642. 10.1016/j.matdes.2012.09.038

[14] Noyan IC, Cohen JB (1987) Residual stress: measurement by diffraction and interpretation. Springer, New York

[15] Jiang WC, Woo W, An GB, Park JU (2013) Neutron diffraction and finite element modeling to study the weld residual stress relaxation induced by cutting. Mater Des 51:415–420. 10.1016/j.matdes.2013.04.053

[16] Sunde M, Serpell LC, Bartlam M, Fraser PE, Pepys MB, Blake CCF (1997) Common core structure of amyloid fibrils by synchrotron X-ray diffraction. J Mol Biol 273(3):729–739. 10.1006/jmbi.1997.1348 9356260 10.1006/jmbi.1997.1348

[17] Chen SL, Tian C (2021) Correction to: recent developments in photoacoustic imaging and sensing for nondestructive testing and evaluation. Vis Comput Ind Biomed Art 4(1):10. 10.1186/S42492-021-00077-x 33914199 10.1186/s42492-021-00077-x PMC8085174

[18] Wang SG, Li YG, Liu CQ, Zhao ZW (2022) An initial residual stress inference method by incorporating monitoring data and mechanism model. Chin J Mech Eng 35(1):82. 10.1186/s10033-022-00746-9

[19] Hu XX, Li YG, Zhao ZW, Liu CQ, Salonitis K (2021) Residual stresses field estimation based on deformation force data using Gaussian process latent variable model. Procedia Manuf 54:279–283. 10.1016/j.promfg.2021.07.044

[20] Zhao ZW, Liu CQ, Li YG, Gao J (2023) A new method for inferencing and representing a workpiece residual stress field using monitored deformation force data. Engineering 22:49–59. 10.1016/j.eng.2022.07.018

[21] Robinson JS, Tanner DA (2008) Reducing residual stress in 7050 aluminum alloy die forgings by heat treatment. J Eng Technol 130(3):031003. 10.1115/1.2931150

[22] Lacarac V, Chang CC, Bramley AN, Tierney MJ, Mcmahon CA, Smith DJ (2004) Predictions and measurements of residual stresses from forging and heat treatment. Proc Inst Mech Eng B J Eng Manuf 218(3):301–313. 10.1243/095440504322984858

[23] Wen T, Liu LT, Huang Q, Chen X, Fang JZ (2018) Evaluation on prediction abilities of constitutive models considering FEA application. J Cent South Univ 25(6):1251–1262. 10.1007/s11771-018-3822-8

[24] Li J, Li FG, Cai J, Wang RT, Yuan ZW, Xue FM (2012) Flow behavior modeling of the 7050 aluminum alloy at elevated temperatures considering the compensation of strain. Mater Des 42:369–377. 10.1016/j.matdes.2012.06.032

[25] Wu DX (2016) Study on processing optimization and quenching residual stresses elimination of long axis 7050 aluminum alloy forgings with H-shape section. Dissertation, Chongqing University

[26] Hao XZ, Li YG, Chen GX, Liu CQ (2018) 6+X locating principle based on dynamic mass centers of structural parts machined by responsive fixtures. Int J Mach Tools Manuf 125:112–122. 10.1016/j.ijmachtools.2017.11.006

